# The efficacy of nonpharmacologic intervention for orthostatic hypotension associated with aging

**DOI:** 10.1212/WNL.0000000000005994

**Published:** 2018-08-14

**Authors:** Julia L. Newton, James Frith

**Affiliations:** From the Institute of Cellular Medicine (J.L.N.), Newcastle University; Falls and Syncope Service (J.L.N., J.F.), Newcastle Upon Tyne Hospitals NHS Foundation Trust; and NIHR Newcastle Biomedical Research Centre (J.F.), Newcastle Upon Tyne Hospitals NHS Trust and Newcastle University, UK.

## Abstract

**Objective:**

To determine the efficacy and safety of nonpharmacologic interventions for orthostatic hypotension (OH) secondary to aging.

**Methods:**

A total of 150 orthostatic challenges were performed in 25 older people (age 60–92 years) to determine cardiovascular responses to bolus water drinking, compression stockings, abdominal compression, and physical countermaneuvers. Primary outcome was response rate as assessed by proportion of participants whose systolic blood pressure (SBP) drop improved by ≥10 mm Hg.

**Results:**

The response rate to bolus water drinking was 56% (95% confidence interval [CI] 36.7–74.2), with standing SBP increasing by 12 mm Hg (95% CI 4–20). Physical countermaneuvers were efficacious in 44% (95% CI 25.8–63.3) but had little effect on standing SBP (+7.5 mm Hg [95% CI −1 to 16]). Abdominal compression was efficacious in 52% (95% CI 32.9–70.7) and improved standing SBP (+10 mm Hg [95% CI 2–18]). Compression stockings were the least efficacious therapy (32% [95% CI 16.1–51.4]) and had little effect on standing SBP (+6 mm Hg [95% CI −1, 13]). No intervention improved symptoms during standing. There were no adverse events.

**Conclusions:**

Bolus water drinking should become the standard first-line nonpharmacologic intervention, whereas compression stockings should be disregarded in this population.

**Classification of evidence:**

This study provides Class III evidence that for older people with OH, bolus water drinking is superior to other nonpharmacologic interventions in decreasing SBP drop.

Orthostatic hypotension (OH) is a disabling condition, resulting from a sustained reduction in blood pressure (BP; ≥20 mm Hg systolic or ≥10 mm Hg diastolic) within 3 minutes of standing.^1^ Aging is one of the most common causes of neurogenic OH, affecting 7% to 30% of community-dwelling older people.^2^ Clinical guidelines recommend nonpharmacologic therapy as first-line treatment in OH, but older people are typically excluded from research, creating a great deal of clinical uncertainty.^3^ Because the older population is expanding rapidly, we can expect a growing demand for evidence in this area.

## Methods

### Population

All participants were >60 years of age and had OH according to international criteria.^1^ Dysautonomia was confirmed on autonomic function testing and was judged to be secondary to aging (in the absence of other identifiable causes). Exclusions were dysphagia, fluid restriction, and inability to wear compression garments. Participants were recruited via the UK Clinical Trials Gateway and a Falls and Syncope Service in Northeast England.

### Setting

Procedures occurred between 9:30 and 11:30 am in the Falls and Syncope Service. Participants refrained from caffeine and nicotine and ate a light breakfast only before attending. Medications were withheld for ≥12 hours before attending.

### Interventions

Selection of nonpharmacologic interventions was based on a recent systematic review and recommendations of the European Federation of Neurological Sciences^3,4^: bolus water drinking (480 mL tap water consumed within 5 minutes), physical countermaneuvers (standing cross-legged^5^), compression stockings (to upper thigh [23–32 mm Hg]), and abdominal compression (elastic belt).

### Procedure

#### Visit 1

To establish a control BP profile, participants rested supine for 10 minutes while undergoing continuous cardiovascular monitoring (Task Force Monitor, CNSystems, Graz, Austria) before standing upright for 3 minutes and noting symptoms. Participants then ingested the water. After 20 minutes, the orthostatic challenge was repeated.^6^

To estimate levels of frailty, dominant handgrip strength was quantified with a hydraulic dynamometer (Jamar, Sammons Preston Inc., Bolingbrook, IL). The Charlson Comorbidity Index score was calculated to illustrate the cohort's comorbidity.

#### Visit 2

A control orthostatic BP profile was established with the aforementioned methods. Participants were randomized to the order in which the interventions were administered by selecting a sealed opaque envelope. An orthostatic challenge (supine and standing BP) was repeated for each intervention with a 20-minute washout period between the challenges.

### Outcomes

The primary outcome was response rate to each intervention (proportion of participants whose systolic BP drop improved ≥10 mm Hg). The secondary outcomes were nadir standing systolic BP, BP drop, adverse events, and symptoms (Orthostatic Hypotension Questionnaire Symptom Assessment^7^: participants rate the severity of 6 symptoms [dizziness/lightheadedness, vision, weakness, fatigue, trouble concentrating, head/neck discomfort] from 0 to 10, from which an average score is derived [maximum severity 10]; the Daily Activity Scale was not evaluated because this is a longer-term measure of symptom impact).

### Analysis

An exact, single-stage, phase 2 study design was used.^8^ The study had 80% power to demonstrate a 30% response rate and a 95% chance of rejecting interventions with response rates ≤10%.

The mean and SD are displayed for normally distributed data; median (range) is used for nonparametric data. Response rates with 95% confidence intervals (CIs) were calculated with the use of exact binomial methods. The paired *t* test and Wilcoxon signed-rank test were used for statistical comparison of secondary outcomes using 2-sided *p* values.

### Standard protocol approvals, registrations, and patient consents

This study was approved by the UK National Research Ethics Service (Newcastle and North Tyneside 2). All participants gave written informed consent. The study was registered prospectively with the UK Clinical Trials Gateway on September 12, 2015 (ISRCTN15084870).

### Classification of evidence

The primary objective was to define the response rate to each therapy. This study provides Class III evidence that for older people with OH, bolus water drinking is superior to other nonpharmacologic interventions in decreasing systolic BP drop.

### Data availability

Anonymized data generated during the current study are available from the corresponding author on reasonable request from individuals affiliated with research or health care institutions.

## Results

**Figure 1:**
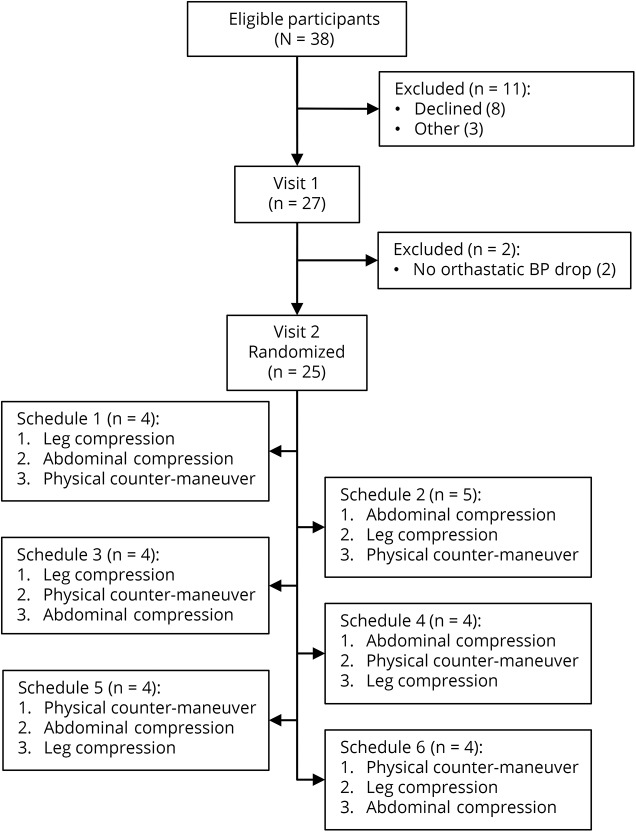
Summary of participant screening and enrollment BP = blood pressure.

**Figure 2:**
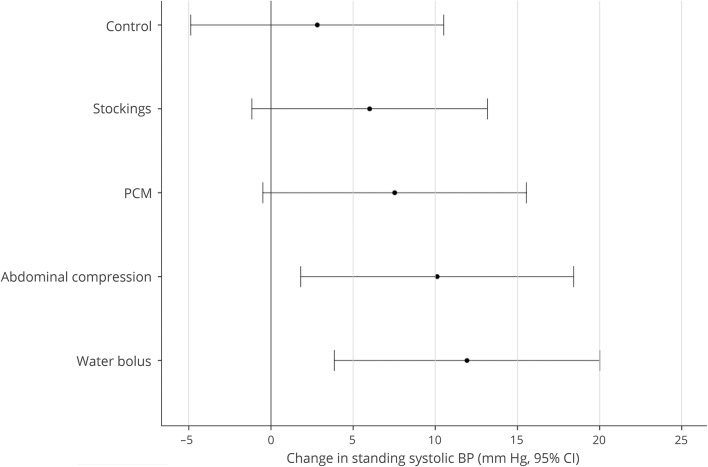
Change in standing systolic BP Change in standing systolic blood pressure (BP) with each intervention compared to no intervention. Control refers to the difference between the baseline postural BP assessments on visits 1 and 2. CI = confidence interval; PCM = physical countermaneuver.

Twenty-five participants were recruited between January and November 2016 (figure 1). Demographic and baseline data are displayed in the table. The median grip strength is slightly lower than UK age-adjusted population norms, suggesting a degree of frailty.^9^ The effect of each intervention on standing systolic BP is shown in figure 2. There were no adverse events. No intervention had a significant impact on the specific symptom of dizziness/lightheadedness (data available from Dryad, table 1, doi.org/10.5061/dryad.h37j22d). Results for the Orthostatic Hypotension Questionnaire Symptom Assessment subscale are provided below.

**Table:**
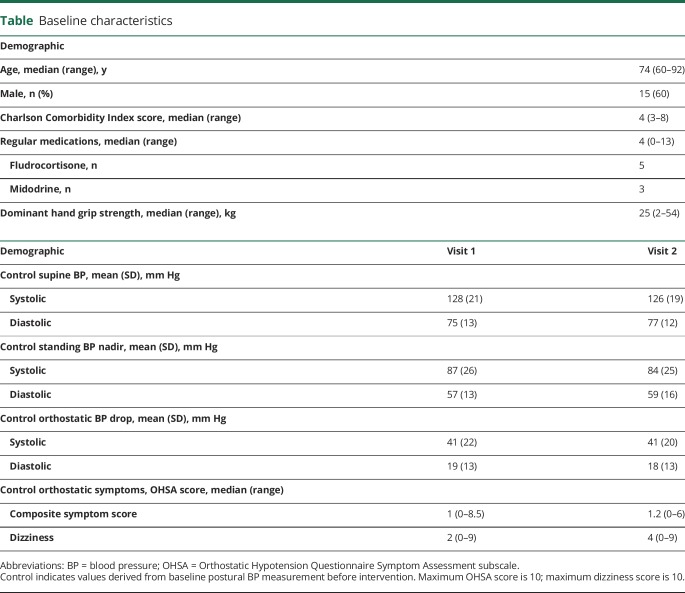
Baseline characteristics

### Bolus water drinking

The median volume of water consumed was 480 mL (248–480 mL). Fourteen participants responded to bolus water drinking (response rate 56%, 95% CI 37%–74%). Systolic BP drop was significantly lower after water (33 [19] mm Hg, *p* = 0.021). There was no effect on diastolic BP drop (15 [12] mm Hg, *p* = 0.103) or on symptoms (1 [0–8], *p* = 0.923).

### Physical countermaneuvers

Eleven participants responded to physical countermaneuvers (response rate 44%, 95% CI 26%–63%). There was no effect on systolic BP drop (35 [24] mm Hg, *p* = 0.085) or on symptoms (2.2 [0–6.8], *p* = 0.117). However, diastolic BP drop improved significantly (13 [16] mm Hg, *p* = 0.047).

### Compression stockings

Eight participants responded to compression stockings (response rate 32%, 95% CI 16%–51%, *p* = 0.002). There was no change in systolic or diastolic BP drop (40 [21] mm Hg, *p* = 0.642 and18 [13] mm Hg, *p* = 0.815, respectively) or in symptoms (0.8 [0–7], *p* = 0.818).

### Abdominal compression

Thirteen participants responded to abdominal compression (response rate 52%, 95% CI 33%–71%, *p* < 0.001). Systolic BP drop reduced significantly (32 [18] mm Hg, *p* = 0.007), but diastolic BP drop did not (15.3 [12] mm Hg, *p* = 0.192). There was no change in symptoms (1.3 [0–6.3], *p* = 0.447).

## Discussion

This study demonstrates that bolus water drinking is the most efficacious nondrug therapy for aging-associated OH. Abdominal compression and physical countermaneuvers also resulted in reasonable response rates but had variable effects on secondary cardiovascular outcomes. In contrast, full leg length compression resulted in relatively low response rates and had no effect on secondary outcomes. The absence of any effect on symptoms is likely explained by a lack of power to detect small changes in secondary outcomes. The median symptom score was much higher during physical countermaneuvers, possibly due to the cardiovascular effects of physical exertion such as vasodilation or possibly to a reduced standing balance.

Because nonpharmaceutical interventions are recommended as first-line therapy and are preferred by older people, it is essential that we develop a robust evidence base for their use,^4^ particularly in the context of the rapidly expanding older population.^10^ Furthermore, there are special considerations that are relevant to older populations. Older people are more likely to have coexisting problems (e.g., urinary incontinence, limited mobility) that may limit the use of nondrug interventions. Compression garments may also be limited by the difficulties of applying and removing these single-handedly. If clinicians are to recommend therapies when barriers exist, it is important that the value of the intervention is known to aid patient education and ultimately adherence.

This phase II study is relatively small, limiting its external validity. Further evaluation is needed to explore the efficacy of combined therapies and to establish long-term effectiveness. It is important to note that in each case, the control orthostatic BP was performed first, closer in time to any preceding meal. This could have exerted greater postprandial hypotensive effects on the control BP compared to the interventions, exaggerating the beneficial effects of the interventions.

Bolus water drinking should become the standard first-line nonpharmacologic intervention, whereas compression stockings should be disregarded in this population.

## Glossary

BP: blood pressure
CI: confidence interval
OH: orthostatic hypotension
